# Transforming respiratory diseases management: a CMO-based hospital pharmaceutical care model

**DOI:** 10.3389/fphar.2024.1461473

**Published:** 2024-10-23

**Authors:** Borja Zarate-Tamames, Noe Garin, Marta Calvin-Lamas, Sonia Jornet, Jose J. Martinez-Simon, Sara Garcia-Gil, Eva M. Garcia-Rebolledo, Ramon Morillo-Verdugo

**Affiliations:** ^1^ Department of Pharmacy, Hospital de la Santa Creu i Sant Pau, Universitat Autònoma de Barcelona, Barcelona, Spain; ^2^ Department of Medicine, Universitat Autònoma de Barcelona, Barcelona, Spain; ^3^ School of Health Science Blanquerna, Universitat Ramon Llull, Barcelona, Spain; ^4^ Department of Pharmacy, Complexo Hospitalario Universitario A Coruña (CHUAC), Instituto de Investigación Biomédica de A Coruña (INIBIC), A Coruña, Spain; ^5^ Departament of Pharmacy, Hospital Universitari de Tarragona Joan XXIII, Tarragona, Spain; ^6^ Department of Pharmacy, Hospital Universitario Fundación Alcorcón, Madrid, Spain; ^7^ Department of Pharmacy, Complejo Hospitalario Universitario de Canarias, Santa Cruz de Tenerife, Spain; ^8^ Department of Pharmacy, Hospital Universitario de Fuenlabrada, Madrid, Spain; ^9^ Department of Pharmacy, Hospital Universitario de Valme, Área de Gestión Sanitaria Sur de Sevilla, Sevilla, España

**Keywords:** pharmaceutical care, respiratory diseases, hospital pharmacy, innovation, behavior, adherence

## Abstract

**Introduction:**

Respiratory diseases encompass a diverse range of conditions that significantly impact global morbidity and mortality. While common diseases like asthma and COPD exhibit moderate symptoms, less prevalent conditions such as pulmonary hypertension and cystic fibrosis profoundly affect quality of life and mortality. The prevalence of these diseases has surged by approximately 40% over the past 3 decades. Despite advancements in pharmacotherapy, challenges in drug administration, adherence, and adverse effects persist. This study aimed to develop and perform an interim validation of a Capacity-Motivation-Opportunity (CMO) model tailored for respiratory outpatients to enhance pharmaceutical care, which is the direct, responsible provision of medication-related care for the purpose of achieving definite outcomes that improve a patient’s quality of life, and overall wellbeing.

**Methodology:**

This cross-sectional, multicenter study was conducted from March 2022 to March 2023. It comprised four phases: 1) forming an expert panel of 15 hospital pharmacists, 2) selecting respiratory pathologies based on prevalence and severity, 3) developing the CMO model’s pillars, and 4) integrating and conducting an interim validation of the model. The Capacity pillar focused on patient stratification and personalized care; the Motivation pillar aligned therapeutic goals through motivational interviewing; and the Opportunity pillar promoted the use of information and communication technologies (ICTs) for telemedicine.

**Results:**

The model included eight respiratory diseases based on expert assessment. For the Capacity pillar, 22 variables were defined for patient stratification, leading to three priority levels for personalized pharmaceutical care. In a preliminary test involving 201 patients across six hospitals, the stratification tool effectively classified patients according to their needs. The Motivation pillar adapted motivational interviewing techniques to support patient adherence and behavior change. The Opportunity pillar established teleconsultation protocols and ICT tools to enhance patient monitoring and care coordination.

**Conclusion:**

The CMO model, tailored for respiratory patients, provides a comprehensive framework for improving pharmaceutical care. By focusing on patient-centered care, aligning therapeutic goals, and leveraging technology, this model addresses the multifaceted needs of individuals with respiratory conditions. Future studies are necessary to validate this model in other healthcare systems and ensure its broad applicability.

## Introduction

Respiratory diseases include a group of conditions known for their extensive heterogeneity, prevalence, symptoms, and morbidity. Certain conditions show high frequency but moderate symptoms and variable mortality rates, such is the case of asthma, COPD, chronic cough or nasal polyposis. In contrast, diseases such as pulmonary hypertension, diffuse interstitial lung disease (ILD), cystic fibrosis and bronchiectasis, are characterized by low prevalence and variable symptomatology, but with a profound impact on quality of life and mortality. In fact, respiratory pathologies are the third leading cause of death in the world (BD Chronic Respiratory Disease Collaborators, 2020). Furthermore, the prevalence of these pathologies has increased in recent years, with an estimated rise of approximately 40% over the past 3 decades, largely due to pollution (Labaki and Han, 2020).

Pharmacotherapy is an essential pillar in the management of these patients. The emergence of new molecules has expanded their therapeutic approach, with a radical improvement in clinical outcomes over the last decades (Charles et al., 2022; Finnerty et al., 2021; McGregor et al., 2019; Pitre et al., 2022; Regard et al., 2022). Despite the improvements in drug treatments, their administration can be challenging due to issues related to administration devices, complex dosing titration, interactions profile, adherence problems, risk of adverse effects, among others, which may benefit from a multidisciplinary approach to ensure optimal health outcomes.

One of the primary challenges in these patients is the compliance with inhalation therapy, which serves as the initial treatment option in numerous cases. The estimated adherence rate to inhalers is approximately 50% (Mäkelä et al., 2013; Valverde-Monge et al., 2022), which poses a significant risk to patients, as poorer adherence has been consistently associated with a higher frequency of exacerbations, increased symptom burden, elevated systemic corticosteroid requirements, more frequent hospital admissions, and elevated disease-related mortality in individuals with asthma or COPD (Belleudi et al., 2016; Garin et al., 2023; Mäkelä et al., 2013; Makhinova et al., 2015; Vestbo et al., 2009; Wiśniewski et al., 2014). Furthermore, another concern is that only one-third of patients correctly perform the inhalation technique (Sanchis et al., 2016). Also, it should be highlighted that the introduction of innovative drug treatments, dispensed to severe patients in the hospital, present a new challenge in their pharmaceutical care.

According to ASHP pharmaceutical care is defined as the direct, responsible provision of medication-related care for the purpose of achieving definite outcomes that improve the patient’s quality of life (American Society of Health-System Pharmacists, 2003). These benefits have been well-demonstrated in conditions like asthma and COPD, especially in the community pharmacy setting (Kim et al., 2021; Makari et al., 2021). Within the hospital environment, several studies have shown positive results on specific respiratory conditions, in the context of research studies which may not necessarily reflect real-life practice. Moreover, there is a need to move from drug-centered to patient-centered models of pharmaceutical care.

**FIGURE 1:**
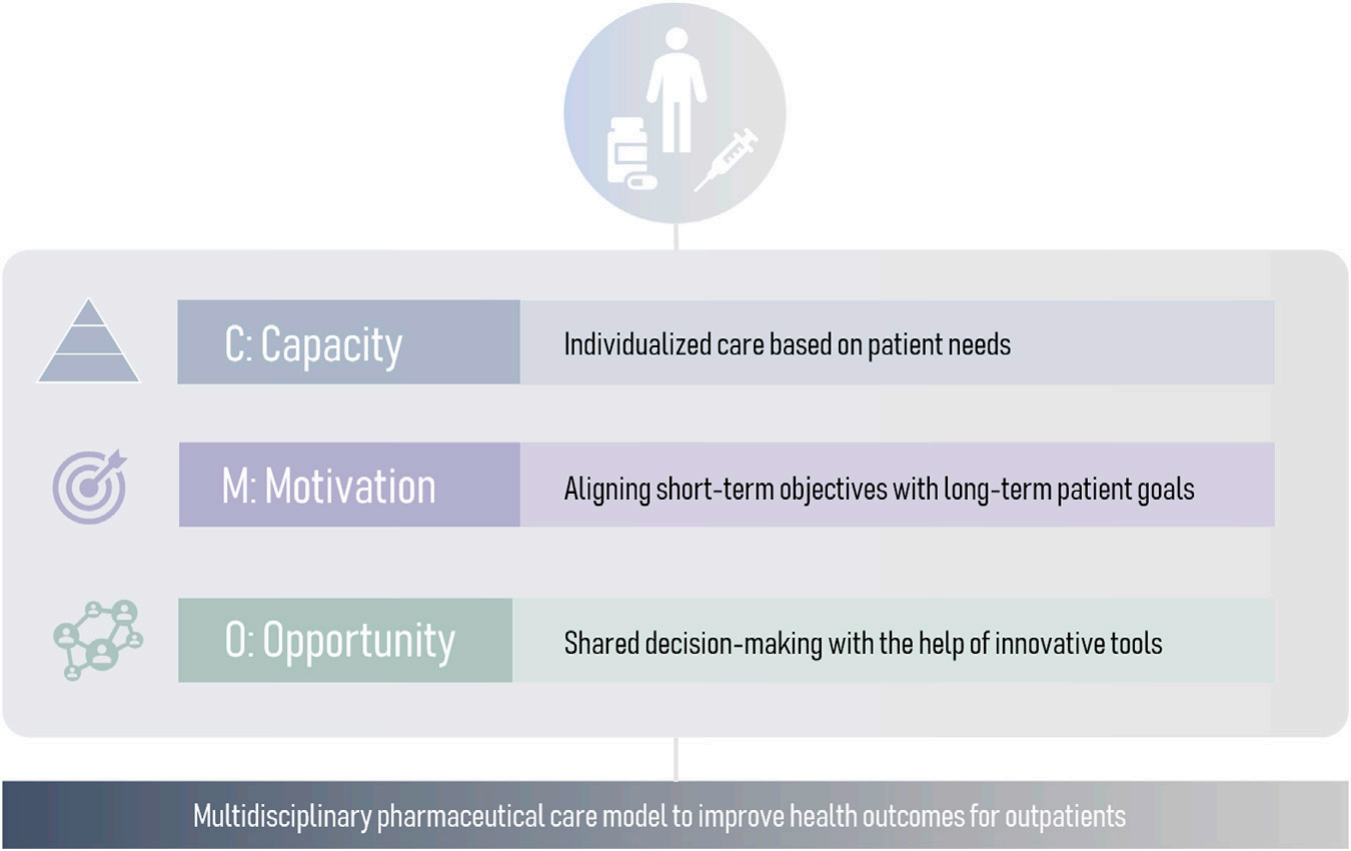
Conceptual framework of the pillars of the CMO model.

Traditional pharmaceutical drug-centered care models focus on adherence, adequate administration and side effect management. While these aspects are essential, this model has become insufficient. A patient-centered model is needed to adapt pharmaceutical care to patient’s needs. With that regard, a novel comprehensive approach is the Capacity-Motivation-Opportunity (CMO) model (Spanish Society of Hospital Pharmacy, 2016), operationalized at three levels: stratification and personalized care (Capacity), therapeutic goal-oriented interventions during clinical interviews (Motivation), and innovative strategies to provide continuous pharmaceutical care (Opportunity) (Figure 1). The CMO model has proven useful in complex diseases such as HIV and rheumatology (Caso-González et al., 2022; Contreras-Macías et al., 2023; Morillo-Verdugo et al., 2022). Moreover, in order to facilitate their implementation, it is crucial to develop versatile models that encompass a wide range of respiratory pathologies.

Thus, the primary objective of this project was to comprehensively develop and perform an interim validation of the CMO model specifically tailored for patients with respiratory conditions. The intention was to establish a robust framework that effectively addresses the multifaceted needs of individuals with respiratory ailments, ultimately aiming to enhance their overall care and wellbeing. By designing and primarily validating the CMO model, this study aimed to contribute to the advancement of patient-centered approaches in respiratory healthcare, facilitating improved clinical decision-making and promoting tailored interventions based on unique capacities, motivations, and opportunities of each individual.

## Materials and methods

This cross-sectional, multicenter study was conducted between March 2022 and March 2023 with the aim of developing and conducting an interim validation of a pharmaceutical care model based on CMO methodology (Spanish Society of Hospital Pharmacy, 2016). The study was conducted in four phases.

**FIGURE 2:**
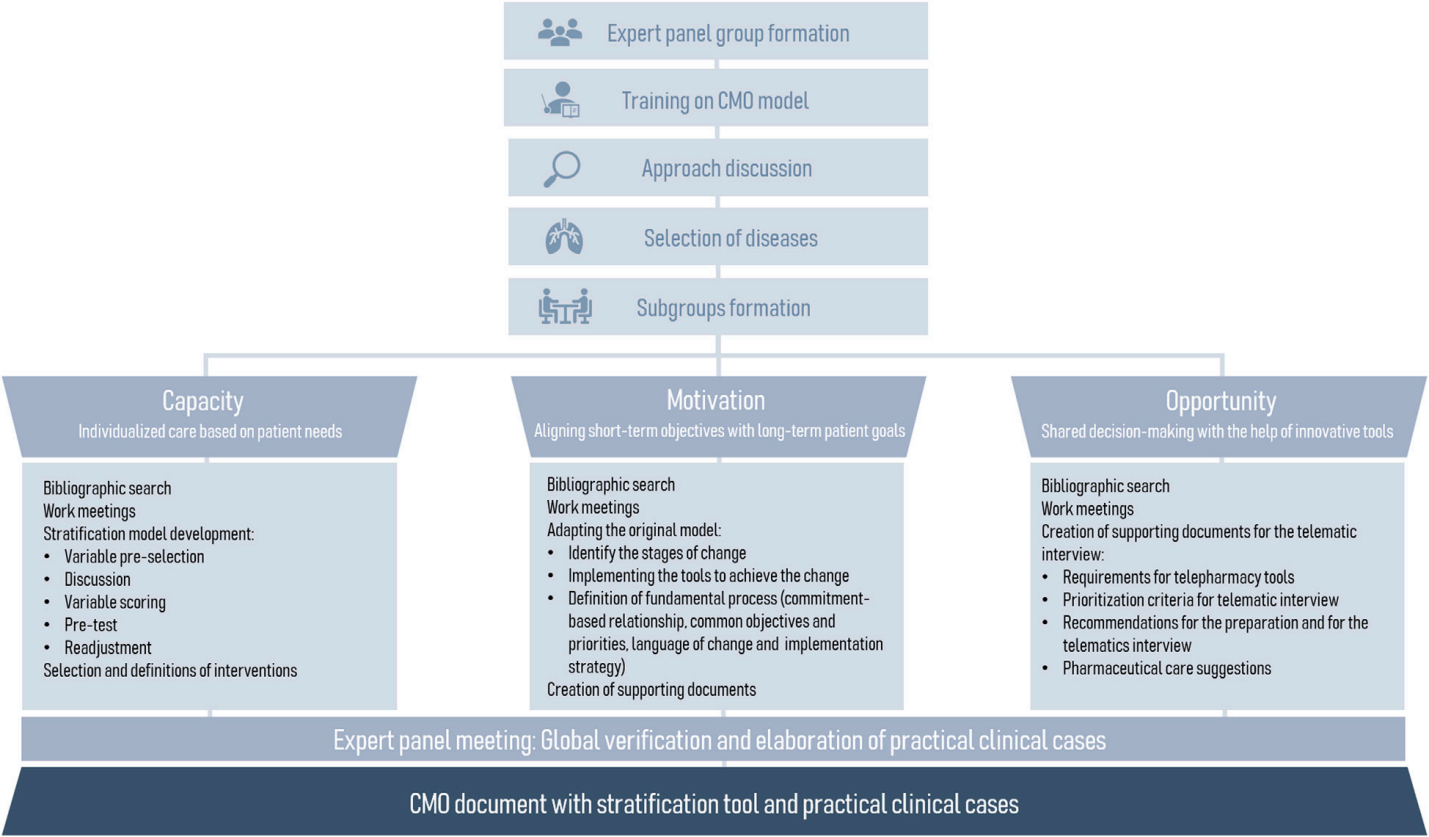
Workflow diagram for the adaptation of the CMO model to respiratory pathologies.

The first phase consisted of the formation of an expert panel consisting of 15 hospital pharmacists: 14 from the respiratory diseases working group of the Spanish Society of Hospital Pharmacy (SEFH) and one expert in CMO methodology. Subsequently, the panel completed a training on the CMO model and the next stages of the study were organized. Also, experts were divided into three workings subgroups according to the three CMO pillars (Figure 2).

In the second phase, during an initial approach discussion, a list of respiratory pathologies to be included in the model was discussed on the basis of a list of potential pathologies compiled by the expert panel. Each group conducted a literature review to gather relevant information, which was shared with the other experts through shared folders for collective analysis. They prepared a comprehensive summary of the scientific evidence available at the time of the study. At the same time, previous CMO models (general CMO model, HIV adaptation, Oncology and Hematology adaptation and chronic patients adaptation) were collected to facilitate their implementation.

The third phase consisted of the development of the pillars of the model by each working subgroup:

1. Capacity pillar: refers to multidisciplinary and patient-centered care, supported by stratification and Pharmaceutical Care (PC) models. The results derived from the preceding bibliographic search were considered, including national and international clinical practice guidelines and other relevant documents, referring to risk factors for respiratory pathologies to reach a consensus on the variables of the stratification tool. Experts reached a consensus on the selection of variables and their corresponding weights in the model. Thereafter the interventions for pharmacotherapeutic monitoring, training and education, and care coordination were defined for each priority level. The cutoff points for priority levels were established based on each patient’s total score. The top 10% of patients with the highest scores were included in Group 1. The next 30% were assigned to Group 2, and the cutoff for Group 3 was determined from the remaining 60% of patients (Craig et al., 1999; Ham et al., 2003; Nuño Solinís, 2007).

To evaluate feasibility, assess stratification capacity, and identify potential areas for improvement, a preliminary test was conducted in six hospitals across the country from 1 July 2022, to 30 September 2022. The sample comprised non-institutionalized adults aged 18 years and above, or those below 18 years if they were attended to in hospital adult outpatient clinics for the management of their respiratory pathologies listed previously. Individuals with severe cognitive decline, with language barriers or needing a legal representative were considered not eligible. Participants were recruited on clinical visits to the Outpatient Pharmacy during the dispensing process or on the administration of medication for respiratory diseases at the day hospital to maximise the access of the pharmacist–patient interaction. Since no established standard for calculating sample size existed in the literature, we decided to include approximately 10% of the patients receiving treatment at each center. Based on data from a national survey in Spain, the outpatient clinic sees 360 patients with respiratory diseases annually (Garin et al., 2024). This translated to a sample size of about 200 patients for this exploratory phase of the study.

All interviewers were experienced hospital pharmacists who had participated in the questionnaire design. The data for all variables except treatment adherence and quality of life were obtained from the electronic health records (Supplementary Appendix SA1). Treatment adherence was measured using the Morisky Green test (Morisky et al., 1986), specific inhaler adherence was assessed with the TAI test (Plaza et al., 2016), and quality of life was evaluated using the EuroQol-5D test (Brooks, 1996), with a short survey questionnaire administered to the participants by specialist clinical pharmacists.

With the final list and weights of the variables, patients were classified into three levels according to their need for pharmaceutical care following a Kaiser Permanente Pyramid model (Feachem et al., 2002). The data evaluation conducted by the expert panel led to refinements in the final model and the identification of common characteristics at each stratification level.

2. Motivation pillar: refers to the alignment of pharmacotherapeutic objectives between the patient and the different healthcare professionals who care for the patient. The pillar was adapted from the main framework, which emphasizes the development of motivational interviewing (MI) and explores the patient’s readiness for change. The most suitable tools for motivational interviewing and its process were then selected, including concepts such as commitment-based relationship, common objectives and priorities, language of change and implementation strategy. This section was adapted to the field of respiratory pathologies from previous CMO-models (Spanish Society of Hospital Pharmacy, 2023; Spanish Society of Hospital Pharmacy, 2020; Spanish Society of Hospital Pharmacy, 2016).
3. Opportunity pillar: focuses on promoting the use of information and communication technologies (ICTs), as an additional strategy to ensure patient health outcomes. In this context, this pillar promotes the use of the telematic interview as an additional appointment to strengthen therapeutic-follow up. The expert panel established the necessary patient characteristics for telepharmacy eligibility based on the patient prioritization model developed by the Spanish Society of Hospital Pharmacy (Spanish Society of Hospital Pharmacy, 2022). The phases for the telematic interview were defined by the expert panel. The PC recommendations to be used in the interviews were gathered and categorized according to their respective pathologies. Additionally, the essential criteria for digital tools to be utilized in telemedicine were discussed and a specific selection was based on the expert opinion derived from their professional expertise. Subsequently, a collection of digital resources and patient organizations related to respiratory diseases management was conducted.

The final phase encompassed the integration of the documents developed in the three pillars, along with their discussion and consensus on the final version. Additionally, four clinical cases were developed to provide practical examples of the model’s application.

The study was approved by the reference Clinical Research Ethics Committee CEI Sevilla SUR (Virgen de Valme University Hospital), Ref: 1664-N-22, 21 September 2022. All investigators worked according to the principles expressed in the Declaration of Helsinki.

## Results

Initially, nine respiratory diseases underwent evaluation for potential inclusion in the model. Following the expert panel’s assessment, eight diseases were selected based on their prevalence, severity, and the accessibility of hospital pharmacists to patients with those conditions (Table 1). The expert panel defined pharmacotherapeutic treatments to include the following: prescribed by the respiratory care physician, medications from specialized or primary care doctors, over-the-counter medicines, and integrative treatments.

**TABLE 1:** List of respiratory diseases included in the model.

Respiratory disease
Asthma (severe)
Cystic fibrosis
Chronic obstructive pulmonary disease^a^
Chronic rhinosinusitis with nasal polyposis
Idiopathic pulmonary fibrosis
Interstitial lung diseases other than idiopathic pulmonary fibrosis^b^
Non-cystic fibrosis bronchiectasis
Pulmonary hypertension

^a^ Includes: alpha-1, antitrypsin deficiency, bronchitis and emphysema.

^b^ Includes: hypersensitivity pneumonitis and silicosis.

### Capacity pillar and stratification risk model

For the development of the stratification tool of the Capacity pillar, 22 consensus variables were defined and divided into five groups: demographic (n = 3), clinical (n = 5), treatment-related (n = 8), socio-sanitary (n = 5) and variables related to the use of healthcare resources (n = 1) (Table 2). Initially, a score was assigned to each variable: five points to the group of demographic variables, 14 to the group of clinical variables, 21 to the treatment-related variables, 16 to the socio-sanitary variables, and three to those related to healthcare resources use.

**TABLE 2:** Description of selected variables included in the stratification tool.

Variable group	Variable	Description
Demographic variables	Pregnancy	Pregnant and *postpartum* patients
	Age	0–15 years (or until the age at which the patient is referred to adult consultation)
		<18 years seen in an adult clinic
		≥65 years
	BMI	Obesity (BMI≥30 kg/m^2^)
		Malnutrition (BMI<18,4 kg/m^2^)
		In patients with cystic fibrosis (BMI<18.4 kg/m2)
Clinical variables	Respiratory comorbidity	Multiple respiratory conditions under treatment
	Non-respiratory comorbidity	Other medical conditions under treatment
	Mental disorders	Mental or behavioral disorders under treatment
	Cognitive impairment	Mild to severe cognitive-sensory impairment
	Severity of the condition	Severe pathology or requiring oxygen therapy (Supplementary Appendix SA4)
Treatment-related variables	Lack of adherence	Considered if the patient is non-adherent to any prescribed medication regardless of whether it is for the respiratory pathology. By MPR, MGL, questionnaire, TAI
	Drugs that can worsen respiratory pathology	Complete list in Supplementary Appendix SA5
	High-alert medicines	Prescribed medicines of the ISMP list
	Pharmacotherapeutic objectives	Pharmacotherapy objectives not reached
	Polypharmacy	>5 drugs
	Complex medicines	Prescription of hospital medications requiring pre-administration handling and/or a medical device
	Naive patient	Naive patient on hospital medication
	Changes in drugs	Changes in drug therapy in the last 6 months
Sociosanitary variables	Tobacco	Smoking
	Alcohol or recreational drugs	Alcoholism and/or drug addiction
	Occupational exposure to particulate matter	Occupational exposure to particulate matter
	Low socio-economic status	Unfavourable socio-economic conditions
	Quality of life	Severe impairment in any of the dimensions of quality of life of the EQ-5D-5L Questionnaire
Healthcare resources variables	Hospitalisations and emergency visits	≥2 admissions and/or 2 visits to the emergency department (primary care and hospital), due to decompensation of the respiratory pathology in the last year

^a^ Use of the paediatric chronic patient model; BMI, body mass index; EQ-5D-5L, 5-level EuroQol-5, dimension questionnaire; ISMP, institute for safe medication practices; MGL, Morisky-Green-Levine; MPR, medication possession ratio; TAI, test of adherence to inhalers.

To evaluate feasibility, assess stratification capacity, and identify potential areas for improvement, a preliminary test was conducted in six hospitals across the country from 1 July 2022, to 30 September 2022. The sample comprised non-institutionalized patients with respiratory patients with respiratory pathology followed at the center. In the preliminary test, a total of 201 patients from six different hospital were evaluated. Four of them were paediatric patients seen in paediatric clinics, so they were excluded from the analysis as they have their own model (Spanish Society of Hospital Pharmacy, 2014).

Three priority levels were established according to the obtained scores, assigning the first level to the patients with highest complexity (Table 3). Level three included 116 (58.9%) patients, level two 61 (31%) patients and level one included 20 (10.1%) patients. The cut-off points for level two and level one was 20 and 31 points, respectively. Pregnant patients (n = 0) or paediatric patients seen in adult consultations (n = 3) were automatically prioritised to level 1 by expert consensus. Following data analysis and discussion, we conducted a reevaluation of variable weighting, leading to modifications in three specific variables. The final score for each group of variables was: five to the group of demographic variables, 14 to the group of clinical variables, 21 to the variables related to treatment, 16 to the socio-sanitary variables, and three to those related to the use of healthcare resources. The distribution and scores for each variable are shown in Table 4.

**TABLE 3:** Description of the sample.

Variable group	Variable	Total (N = 197^a^)	P1 (N = 21)	P2 (N = 64)	P3 (N = 112)
Demographic variables	Age (n, %) 18–64 years ≥65 years	107 (54) 90 (46)	9 (43) 12 (57)	28 (44) 36 (56)	70 (63) 42 (37)
	BMI (n, %) BMI<18,4 kg/m2 BMI 18.4-29.9 kg/m2 BMI≥30 kg/m2	4 (2) 149 (76) 44 (22)	2 (5) 9 (47) 10 (48)	1 (2) 47 (73) 16 (25)	1 (1) 93 (83) 18 (16)
Clinical variables	Multiple respiratory conditions under treatment (n, %)	73 (37)	9 (43)	30 (47)	34 (30)
	Other medical conditions under treatment (n, %)	139 (71)	20 (95)	59 (92)	62 (55)
	Mental or behavioral disorders under treatment (n, %)	22 (11)	6 (29)	8 (13)	8 (7)
	Mild to severe cognitive-sensory impairment (n, %)	14 (7)	4 (19)	7 (11)	3 (3)
	Severe pathology or requiring oxygen therapy^b^ (n, %)	107 (54)	19 (90)	48 (75)	40 (36)
Treatment-related variables	Non-adherent to any prescribed medication (n, %)	71 (36)	16 (76)	28 (44)	27 (24)
	Drugs that can worsen respiratory pathologies (n, %)	21 (11)	9 (43)	7 (11)	5 (4)
	High-alert medicines (n, %)	91 (46)	18 (86)	45 (70)	28 (25)
	Pharmacotherapy objectives not reached (n, %)	66 (34)	15 (71)	26 (41)	25 (22)
	Polypharmacy (>5 drugs) (n, %)	147 (75)	19 (90)	63 (98)	65 (58)
	Prescription of hospital medications requiring pre-administration handling and/or a medical device (n, %)	135 (69)	17 (81)	47 (71)	71 (63)
	Naive patient on hospital medication (n, %)	69 (36)	14 (67)	23 (36)	32 (29)
	Changes in drug therapy in the last 6 months (n, %)	103 (52)	15 (71)	34 (53)	54 (48)
Sociosanitary variables	Smoking (n, %)	20 (10)	7 (33)	6 (9)	7 (6)
	Alcoholism and/or drug addiction (n, %)	4 (2)	1 (5)	2 (3)	1 (1)
	Occupational exposure to particulate matter (n, %)	14 (7)	5 (24)	5 (8)	4 (4)
	Unfavourable socio-economic conditions (n, %)	7 (4)	3 (14)	3 (5)	1 (1)
	Severe impairment in any of the dimensions of quality of life of the EQ-5D-5L Questionnaire. (n, %)	96 (49)	16 (76)	49 (77)	32 (29)
Healthcare resources variables	≥2 admissions and/or 2 visits to the emergency department (primary care and hospital), due to decompensation of the respiratory pathology in the last year. (n, %)	70 (36)	16 (76)	30 (47)	24 (21)

^a^ A total of 201 patients were included. Frequency and proportions are presented for 197 adults. The remaining four patients are pediatric cases, prioritized without additional variables.

^b^ As described in Supplementary Appendix SA4.

BMI, body mass index; EQ-5D-5L, 5-level EuroQol- 5, Dimension Questionnaire, P1, Priority group 1; P2, priority group 2; P3, Priority group 3.

**TABLE 4:** Description and final score for the variables included in the stratification tool.

Variable group	Variable	Description	Score
Demographic variables	Pregnancy	Pregnant and *postpartum* patients	Priority 1
	Age	0–15 years (or until the age at which the patient is referred to adult consultation)	^a^
		<18 years seen in an adult clinic	Priority 1
		≥65 years	2
	BMI	Obesity (BMI≥30 kg/m^2^)	3
		Malnutrition (BMI<18,4kg/m^2^)	1
		In patients with cystic fibrosis (BMI<18.4 kg/m^2^)	3
Clinical variables	Respiratory comorbidity	Multiple respiratory conditions under treatment	2
	Non-respiratory comorbidity	Other medical conditions under treatment	3
	Mental disorders	Mental or behavioral disorders under treatment	3
	Cognitive impairment	Mild to severe cognitive-sensory impairment	4
	Severity of the condition	Severe pathology or requiring oxygen therapy (Supplementary Appendix SA4)	2
Treatment-related variables	Lack of adherence	Considered if the patient is non-adherent to any prescribed medication regardless of whether it is for the respiratory pathology. By MPR, MGL, questionnaire, TAI	4
	Drugs that can worsen respiratory pathology	Complete list in Supplementary Appendix SA5	4
	High-alert medicines	Prescribed medicines of the ISMP list	4
	Pharmacotherapeutic objectives	Pharmacotherapy objectives not reached	2
	Polypharmacy	>5 drugs	3
	Complex medicines	Prescription of hospital medications requiring pre-administration handling and/or a medical device	1
	Naive patient	Naive patient on hospital medication	1
	Changes in drugs	Changes in drug therapy in the last 6 months	1
Sociosanitary variables	Tobacco	Smoking	4
	Alcohol or recreational drugs	Alcoholism and/or drug addiction	3
	Occupational exposure to particulate matter	Occupational exposure to particulate matter	3
	Low socio-economic status	Unfavourable socio-economic conditions	3
	Quality of life	Severe impairment in any of the dimensions of quality of life of the EQ-5D-5L Questionnaire	3
Healthcare resources variables	Hospitalisations and emergency visits	≥2 admissions and/or 2 visits to the emergency department (primary care and hospital), due to decompensation of the respiratory pathology in the last year	3

^a^ Use of the paediatric chronic patient model; BMI, body mass index; EQ-5D-5L, 5-level EuroQol-5, dimension questionnaire; ISMP, institute for safe medication practices; MGL, Morisky-Green-Levine; MPR, medication possession ratio; TAI, test of adherence to inhalers.

The score was recalculated for all patients. Finally, the new priority groups and cut-off points were reestablished: level three included 112 (56.8%) patients, level two 64 (32.5%) patients and level one included 21 (10.7%) patients. The cut-off points for level two and level one increase to 21 and 32 points, respectively. Pregnant patients (n = 0) or paediatric patients seen in adult consultations (n = 3) were automatically prioritised to level 1.

The most prevalent problems found in the sample were: polypharmacy (defined as the regular use of five or more chronic drugs) (75%), presence of non-respiratory comorbidities (71%), presence of hospital drugs requiring previous manipulation (69%), high severity degree (54%), last 6-month pharmacotherapy changes (52%). The most common variables in each group were: in group 1 the presence of non-respiratory comorbidities (95%), polypharmacy (90%) and the presence of severe illness or requiring oxygen therapy (90%). In group 2 polypharmacy (98%), the presence of non-respiratory comorbidities (92%) and the significant decrease or severe impairment in any of the dimensions of the EQ-5D-5L Quality of Life Questionnaire (77%). Finally, for group 3, polypharmacy (58%), presence of non-respiratory comorbidities (55%) and the age of the patient (37%) (Table 3).

**FIGURE 3:**
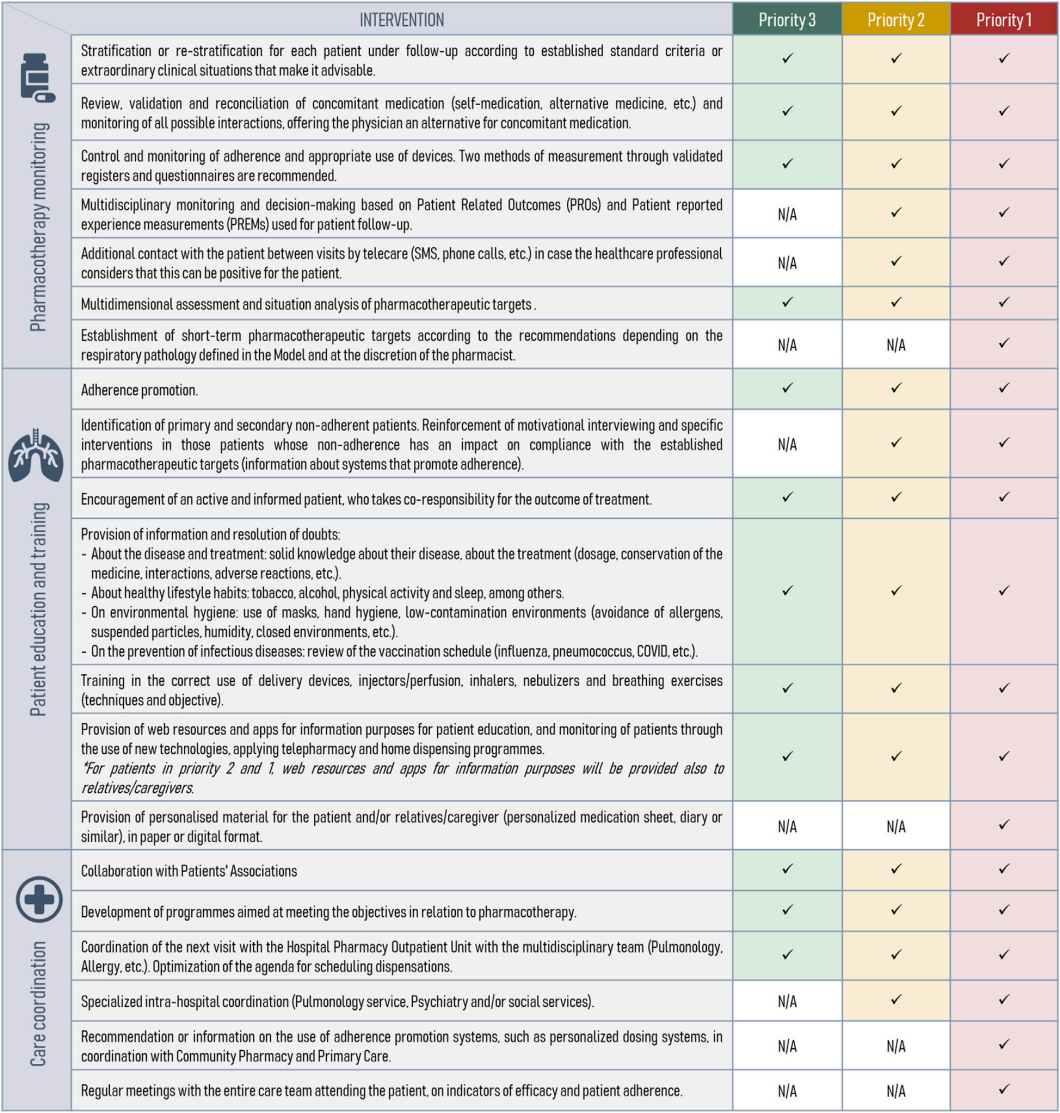
Categorization and description of individualized pharmaceutical care interventions by priority levels. N/A, not applicable.

Subsequent to the stratification tool’s development, the expert panel defined specific PC interventions at each severity level to deliver optimal individualized care. Conceptually, these interventions fell into three main areas of action (Figure 3):

• Pharmacotherapeutic monitoring (n = 7): review of the appropriateness, effectiveness and safety of treatments.
• Patient training and education (n = 7): information on medication, support for the administrative processing of treatments, and promotion of co-responsibility in the outcome of treatment.
• Care coordination (n = 6): Development of protocols, guidelines, and Standard Operating Procedures (SOPs) to unify criteria among healthcare professionals, such as pulmonologists and social services, ensuring coordinated care across different healthcare levels. This includes enhancing documentation practices through the integration of Electronic Health Records (EHR) to support seamless communication and continuity of patient care.

**FIGURE 4:**
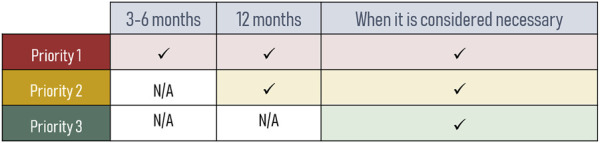
Monitoring frequency by priority levels.

These interventions are cumulative, meaning that priority level 1 encompasses interventions from the preceding two levels (Figure 3). Additionally, the monitoring frequency was determined based on the priority level (Figure 4).

In order to facilitate pharmaceutical interventions at all priority levels, various support tools were proposed: a) dual PC: Telepharmacy and Mobile Health (Spanish Society of Hospital Pharmacy, 2022) b) bidirectional communication tools and c) use of Standard Operating Procedures (SOP) and recording interventions in the electronic medical records (Supplementary Appendix SA2).

### Motivation pillar

An expert panel adapted this pillar, which focused on the implementation of MI to facilitate transformative change, from the original CMO model. The primary objective was to provide support regarding the identification and resolution of ambivalence, characterized by the simultaneous desire for change and resistance to change, as well as the orientation towards the change process. While a comprehensive understanding of the classic model of the patient’s change journey defined by Prochaska and DiClemente (Prochaska and DiClemente, 1982) which includes the stages of pre-contemplation, contemplation, preparation, action, maintenance, and relapse, is essential, we know that in practice, the patient may move to action from any stage. Detecting the patient’s current stage is crucial to evaluate and focus the intervention, either to initiate the process or to work towards a possible future start. To attain the objective of tipping the balance towards change, the application of MI tools was introduced this section:

• Open Questions: facilitating patients to articulate their thoughts and concerns.
• Affirmations: enhancing patient’s self-confidence.
• Reflective Listening: Enabling the expression of empathy and an understanding of the patient’s perspective.
• Summaries: emphasizing change goals by selectively condensing the reasons for change (among other functions).

The expert panel defined four fundamental processes in the MI, described briefly herein. First and foremost, the establishment of a commitment-based relationship built on mutual respect and trust. In this context, it was recommended to provide a consultation environment where conflicts can be safely explored and challenging realities can be addressed. Consequently, it was deemed essential that the consultations conducted by the hospital pharmacist be private, allowing adequate time for each patient. Secondly, once a relationship of trust had been established, it was necessary to set an agenda that includes both the patient’s and the practitioner’s objectives and priorities. This would allow focus and direction to be maintained. The third process was to enable the patients to express their own reasons for change. This was identified through the “language of change.” To achieve this, we must assist the patient in discovering and acknowledging their own motivation. The fourth process was to develop a specific and agreed plan for implementing the change. This plan should be specific, measurable, achievable, relevant and time-bound. It was recognised as essential to plan care visits, establish a daily patient care schedule, and have a comprehensive understanding of the stratification level to effectively implement MI to its fullest extent.

Throughout this process, the resulting Motivational pillar also emphasized the importance of fundamental principles of MI, such as collaboration, evocation, compassion and respect for autonomy. Additionally, it is crucial to recognize what to avoid, including neglecting nonverbal communication, improvising, showing a deficit or excess of emotion, not listening, neglecting time, or showing arrogance.

### Opportunity pillar

**FIGURE 5:**
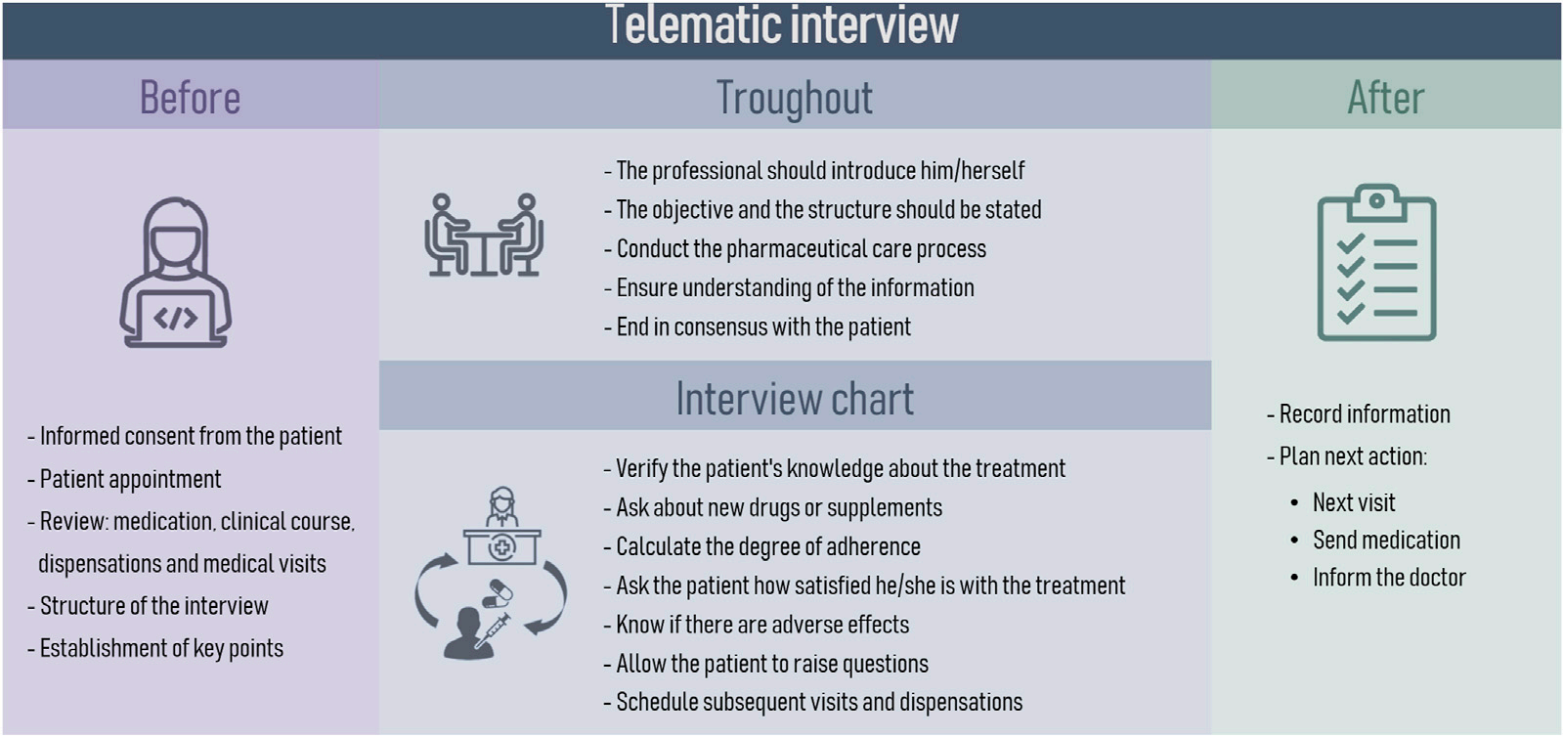
Phases and organizational structure of the telematic interview.

Recommendations for teleconsultation were carefully chosen by the expert panel from various clinical guidelines. The phases of the telematic interview were defined as well as the organizational structure to be implemented during the PC (Figure 5). Taking into account the criteria of the national guideline on telepharmacy developed by the SEFH (Spanish Society of Hospital Pharmacy, 2022), the characteristics to be taken into account for the digital tools used in telepharmacy were described (Table 5). To facilitate interventions during telepharmacy, a summary of recommendations from the SEPAR guidelines on teleconsultancy was made (Table 6) (Díaz Lobato et al., 2022).

**TABLE 5:** Characteristics to be considered in the selection of digital tools used for telepharmacy.

Area	Description
Integration	Integration into health information systems
Maintenance	Guaranteed maintenance
Training	Appropriate training plan for staff involved in training for this service
Proactive risk assessment	Risk mitigation strategies (e.g., for damaged equipment, helpdesks, software protection, virus protocols, hacking, etc.)
Update	Plan for periodic reassessment of technology solutions available on the market and their suitability for the stated clinical objective
User experience	Plan to periodically evaluate the experience of users (professionals and patients) and implement the improvements detected
Support service	Plan to establish how the user service will be carried out for training and resolution of doubts and incidents

**TABLE 6:** Characteristics to be considered in the selection of digital tools used for telepharmacy.

Respiratory disease	Recommendation
Asthma	- Checking adherence to inhalers: TAI - Reinforcing inhaler technique: SEPAR’s ForoAsma; GEMA Inhalapp, Inhalers and Inhalchek^®^ (requires registration by the pharmacist)
Bronchiectasis	- Review the aerosol therapy technique using one of the following platforms: SEPAR’s ForoAsma GEMA Inhalapp, Inhalers and Inhalchek (requires registration by the registration by the pharmacist) - Vaccination reminder (flu and pneumococcal)
COPD	- Review of smoking habits - Reinforce physical exercise (30 min/day) - Check compliance with non-pharmacological treatment (oxygen therapy, vaccination) - Carry out the TAI questionnaire - Reinforce inhalation technique: SEPAR’s ForoAsma; GEMA Inhalapp, Inhalers and Inhalchek (requires registration by the pharmacist)
Idiopathic pulmonary fibrosis	- Offer non-pharmacological measures: diet, sun protection and probiotics - Vaccination reminders (flu and pneumococcal) - Thoroughly review side effects
Cystic Fibrosis	- Remind patients to monitor their pulse, weight and height - Encourage proper adherence to treatment - Check for proper inhalation technique and correct hygiene of the material used - Encourage non-pharmacological measures: use of masks, hand washing, avoidance of enclosed areas and proper nutrition
Pulmonary hypertension	- Reminder of the need for contraceptive use in fertile women - Reinforce adherence to concomitant treatment
Pulmonary embolism	-Ensure correct administration of anticoagulants and anticoagulants, as well as INR monitoring - Encourage smoking cessation
Silicosis	- To assess the degree of motivation to quit smoking - Offer advice on how to control withdrawal symptoms: irritability, increase in appetite, insomnia
Tuberculosis	- Monitor toxicity: fever, visual, digestive or skin disorders, etc - Review the administration schedule - Reinforce adherence to treatment

COPD, chronic obstructive pulmonary disease; GEMA, spanish guideline on the management of asthma; INR, international normalized ratio; SEPAR, spanish society of pneumology and thoracic surgery; TAI, test of adherence to inhalers.

**FIGURE 6:**
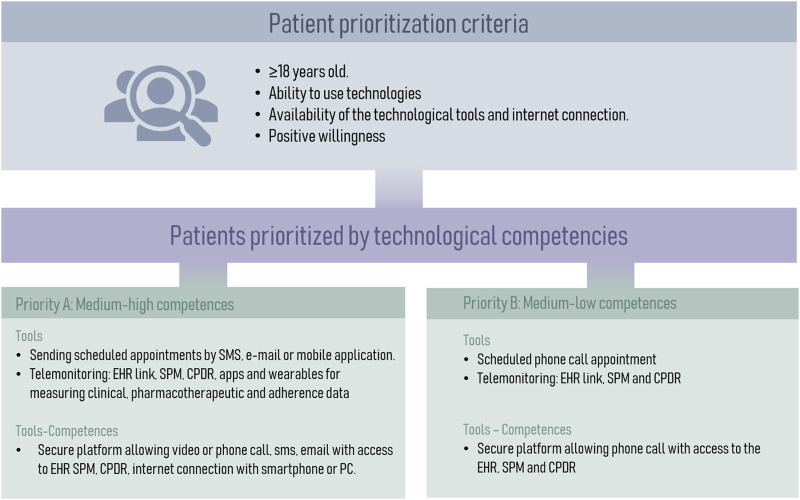
Categorization of Patients for Telemedicine Based on Digital Skills. CPDR, community pharmacy dispensing records; EHR, electronic health records; SPM, single prescription module.

To prioritize patients most suitable for telemedicine, it was decided to categorize them into two groups (Figure 6). Group A comprises high-priority patients who possess advanced digital skills and the capability to utilize various tools available for telemedicine. On the other hand, Group B consists of patients with limited digital proficiency but possess the potential to adapt to new technologies.

Finally, a search was carried out of different information resources available to provide the professionals and the foundations and associations available to patients (Supplementary Appendix SA3).

## Discussion

To the best of our knowledge, this is the first pharmaceutical care model specifically tailored for the outpatient care of patients with respiratory pathologies in the hospital setting at a national level. This approach is based on a global patient perspective, placing the patient at the center of care and ensuring comprehensive and specialized care, and encompass the main respiratory diseases attended at this level of care.

In the past, various models of pharmaceutical care have been developed for different pathologies and at different care levels, leading to improvement in the quality of patient care received (Chung et al., 2014; Jaber et al., 1996; Li et al., 2020; Marfo and Owusu-Daaku, 2017; Wang et al., 2023). However, despite being useful tools, they have numerous limitations that have become more pronounced in recent years due to the advances of new technology, the evolving needs of patients, and the transformation of the professional-patient relationship, which has shifted the focus from the disease to the patient (Boivin et al., 2022; Cen et al., 2022; Park et al., 2022). This shift is vital, serving as a disruptor to break through professional inertia (Codsi et al., 2021).

The vast majority of published models are based on specific pathologies or subpopulation groups (Almahdi et al., 2020; Chung et al., 2014; Jaber et al., 1996; Li et al., 2021; 2020; Marfo and Owusu-Daaku, 2017; Wang et al., 2023; Wermeille et al., 2004). This requires the development of a model for each pathology and specific training for healthcare professionals for each of them, making their implementation challenging. Additionally, patients are often affected by multiple pathologies, a situation that is sometimes overlooked but necessary to consider to provide an adequate pharmaceutical care. Among the models developed in recent years, one of the most interesting has been the CMO model. This model has been shown to improve health outcomes such as medication adherence, mortality and patient experience in certain groups of diseases (Caso-González et al., 2022; Contreras-Macías et al., 2023; Morillo-Verdugo et al., 2022).

Our model addresses a series of pathologies (Table 1) in an innovative way, sharing a common foundation but with distinct nuances across these diseases. Unlike conventional approaches, the model shifts the focus from the pathology to the individual. Many of the efforts available so far focused on specific diseases, mostly within a research environment, making standardization challenging and compromising their practical viability and limited application, as it would not be feasible to have an individual model for each pathology (Basheti et al., 2019; Bedouch et al., 2011; Duwez et al., 2020; Lin et al., 2022; Nguyen et al., 2019; Tommelein et al., 2014; Wei et al., 2014). As a result, we developed a versatile tool, based on three pillars (Capacity-Motivation-Opportunity) ready to be easily applicable in daily medical practice. This model advocates longitudinal monitoring, contrasting with the regular clinical practice (Morillo-Verdugo et al., 2022; Spanish Society of Hospital Pharmacy, 2016).

The capacity pillar of our model allows patients to be identified and classified using a stratification tool to subsequently provide personalized PC adapted to the needs of individual patients (Figure 4). Stratification tools allow the application of standardized pharmaceutical intervention strategies, appropriate to each of the established risk levels (Figure 3), and have been previously associated with positive health outcomes (Cantillana-Suárez et al., 2021; Contreras-Macías et al., 2023; Guzmán Ramos et al., 2021; Martínez Sesmero et al., 2024). Consequently, we could focus on those patients who will benefit the most from pharmacist interventions. However, to ensure feasibility it is preferable that the stratification is automatic or semi-automatic in order to spend as little time as possible to perform it. Most of the 22 variables (Table 2) used in our stratification tool can be automatized or easily obtained from clinical records or patients, which may facilitate the implementation of the tool in the clinical practice.

The motivation pillar aims to help patients in their process of change. In a comprehensive meta-analysis including 119 studies, motivational interviewing was found to produce significant positive effects across a varied group of problem domains, including adherence to medication, smoking or physical health. For example, MI is related to increase medication adherence in diabetes mellitus or HIV (Christie and Channon, 2014; DiIorio et al., 2008; Powell et al., 2014). In the field of respiratory diseases, the use of MI not only is suggested to improve adherence in pilot studies (Lavoie et al., 2014; Naderloo et al., 2018; Taheri et al., 2023), but also other relevant health variables such as self-efficacy, quality of life, physical activity, hospitalization or perception of lung function (Feldman et al., 2023; Rausch Osthoff et al., 2021; Rehman et al., 2017; Wang et al., 2022). However, this activity is rarely included in standardized pharmaceutical care models beyond the context of research studies. In motivational interviewing, its correct application is fundamental for the correct development of the model. An inexperienced pharmacist may find it difficult to detect all the communication barriers and to identify when the patient is ready to change. Other barriers for the implementation of motivational interviewing in the respiratory field are the feeling of professional already doing this activity, feeling different to their usual style of working or eliminating those suppressing behaviors antagonist to this type of intervention (Shannon et al., 2017). This is perhaps the most difficult pillar to master completely. However, its development will allow us to substantially increase the success of our interventions by aligning objectives with the patient.

The opportunity pillar enables the application of technological development (Figure 6) in the field of pharmaceutical care. The gradual acceptance of new technologies by professionals and patients will facilitate the global implementation of telepharmacy in the future. Despite current limitations such as privacy or the low level of digital literacy of patients (Unni et al., 2021), telepharmacy has proven to be useful in some pathologies (Cao et al., 2022; Martínez-Santana et al., 2021) and has managed to democratise access to pharmaceutical care, allowing it to reach developed rural areas (Morillo-Verdugo et al., 2023; Nwachuya et al., 2023). Its application in patients with respiratory pathologies, coupled with the recent development of telemonitoring sensors in inhalers, which have already proven useful (Garin et al., 2023), bodes well for development in this area.

This study has some limitations. The CMO model is a broad tool that requires a learning period to develop its full potential. However, the integration of increasingly widespread IT tools, such as the stratification tool, could facilitate the practical implementation of this model. Despite the initial efforts needed for its implementation, once established, it can be applied to a large number of patients as it covers different respiratory diseases. A limited number of hospitals participated in the development of this model. However, their characteristics and geographical distribution is representative of the vast majority of hospitals in our region. Technological tools are essential for the correct development of certain pillars, such as the Opportunity pillar. Although not indispensable, they can also assist the Capacity pillar by facilitating patient stratification. Future technological advancements in hospital information systems may facilitate the implementation of the model in daily practice. Also, while the CMO model is already being used in clinical practice in respiratory diseases, we have planned an implementation study. This study will analyze the model’s potential, benefits, and limitations in clinical practice and provide insight into its effectiveness. The results of this analysis will guide further adaptations of the model and support its broader application in respiratory care. In the future, additional studies will be necessary to validate the model in healthcare systems from other countries.

This study introduces a new pharmaceutical care model tailored for outpatient respiratory patients at the hospital level, focusing on patient-centered care. The CMO model addresses patient needs through three pillars: Capacity (patient stratification and personalized care), Motivation (motivational interviewing to improve adherence), and Opportunity (telepharmacy and digital tools for continuous care). Our findings show that the CMO model is feasible and potentially of great interest. The Capacity pillar effectively prioritizes patients for tailored interventions (Figure 3). The Motivation pillar aims to enhance patient engagement and adherence, while the Opportunity pillar highlights the potential of telepharmacy and digital healthcare technologies. Despite requiring initial effort for implementation, the model promises significant improvements in clinical outcomes and patient quality of life. Future studies are needed to validate the model in different healthcare settings and ensure its broad adoption. Overall, the CMO model represents a significant step forward in respiratory patient care.

## Data Availability

The datasets presented in this article are not readily available because The results presented in this article mainly refers to the methodological development and interim validation of a pharmaceutical care model. Requests to access the datasets should be directed to Ramon Morillo, ralejandro.morillo.sspa@juntadeandalucia.es.
