# Supplementary material for: Transforming respiratory diseases management: a CMO-based hospital pharmaceutical care model
Source: Front Pharmacol. 2024 Oct 23;15:1461473. doi: 10.3389/fphar.2024.1461473 (PMC11540901; doi:10.3389/fphar.2024.1461473)
Supplement: Supplementary file 1 [file DataSheet2.PDF]

Support tools for pharmaceutical interventions

Dual Pharmaceutical Care (PC) - Telepharmacy and Mobile Health

The use of mobile devices and global networks will be promoted where possible and whenever deemed necessary to provide where possible and whenever deemed necessary, to offer information and assistance services at a distance in complementary to face-to-face care, either synchronously or asynchronously.

- Mobile phones (SMS or Multimedia Messaging Service (MMS))
- Websites and applications (recommended in the Opportunity pillar)
- Smart Devices (wearables and other health technologies)

Bidirectional communication tools

The development and use of communication channels and tools between the different healthcare professionals involved at all levels of care (between hospital care, primary care and community pharmacy) will be encouraged in order to unify criteria and consultations between professionals on how to deal with adverse reactions and resolve possible incidents.

Standard Operating Procedures (SOP) and recording interventions in the electronic medical records

In each Hospital Pharmacy Department, there will be standard operating procedures that serve as guidelines for the activities to be carried out and that ensure the quality and standardization of the process. In addition, pharmaceutical actions must be recorded in the patient's clinical history. In this regard, it is recommended that a taxonomy be developed to standardize the recording of interventions.
