# Supplementary material for: Transforming respiratory diseases management: a CMO-based hospital pharmaceutical care model
Source: Front Pharmacol. 2024 Oct 23;15:1461473. doi: 10.3389/fphar.2024.1461473 (PMC11540901; doi:10.3389/fphar.2024.1461473)
Supplement: Supplementary file 2 [file DataSheet4.PDF]

Disease severity definitions

Disease	Definition of severe disease
Severe asthma	- Patient with Severe Uncontrolled Asthma
Pulmonary Hypertension	- Functional class III or IV
COPD	- Patient with COPD GOLD E (GOLD guidelines)<br>- Patient is stratified as high risk according to the GesEPOC guidelines
Chronic rhinosinusitis with nasal polyposis	-VAS >7 or SNOT-22 >50
Idiopathic pulmonary fibrosis	- FVC <50 %<br>- DLCO <30%<br>- Absolute reduction in FVC ≥10% in the last 6-12 months<br>- Absolute reduction in DLCO ≥15% in the last 6-12 months
Cystic Fibrosis	-FEV1 <40%
Non-cystic fibrosis bronchiectasis	- Clinical worsening or progression as assessed by the responsible healthcare provider<br>- BSI > 8<br>- FACED > 5<br>- CAT > 21

BSI: Bronchiectasis severity index; CAT: COPD assessment test; COPD: Chronic obstructive pulmonary disease; DLCO: Diffusing capacity of the lungs for carbon monoxide; FEV1: Forced expiratory volume; FVC: Forced vital capacity, SNOT-22: 22-item Sinonasal outcome Test; VAS: visual analogue scale

Severe uncontrolled asthma:

- Patient with severe uncontrolled asthma, defined as asthma disease that remains poorly controlled despite the treatment with a combination of IGC/LABA, at high doses in the last year, or oral glucocorticoids for at least 6 months of the same period.

Lack of control will be considered as lack of control if any of the following characteristics are met:

- Asthma Control Test (ACT) < 20 or Asthma Control Questionnaire (ACQ) > 1.5.
- ≥ 2 severe exacerbations or having received ≥ 2 cycles of oral glucocorticoids (of ≥ 3 days each) in the previous year.
- ≥ 1 hospitalisation for severe exacerbation in the previous year.
- Chronic airflow limitation (forced expiratory volume in the first second/forced vital capacity [FEV1/FVC] ratio < 0.7 or FEV1 < 80 % of predicted) after use of appropriate treatment (provided the best FEV1 is greater than 80 %).
- Requires relief/rescue medication (short-acting β2-adrenergic agonist) more than 2 days a month.

Pulmonary Hypertension - WHO functional class:

- Class I: Patients with PH who have no limitation in usual physical activity, regular physical activity does not cause an increase in dyspnea (shortness of breath), fatigue, chest pain, or presyncope (fainting).
- Class II: Patients with PH who have a slight limitation in physical activity. They are comfortable at rest, but normal physical activity causes an increase in dyspnea, fatigue, chest pain, or presyncope.
- Class III: Patients with PH who have a marked limitation in physical activity. They are comfortable at rest, but less than ordinary activity causes an increase in dyspnea, fatigue, chest pain, or presyncope.
- Class IV: Patients with PH who are unable to carry out any physical activity without symptoms and may have signs of right ventricular failure at rest. Dyspnea and fatigue may be present at rest, and symptoms are increased by any physical activity.
