# Supplementary material for: Transforming respiratory diseases management: a CMO-based hospital pharmaceutical care model
Source: Front Pharmacol. 2024 Oct 23;15:1461473. doi: 10.3389/fphar.2024.1461473 (PMC11540901; doi:10.3389/fphar.2024.1461473)
Supplement: Supplementary file 3 [file DataSheet3.PDF]

Digital health resources and apps available to patients

Web	App	X (Twitter)
www.separcontenidos.es<br>www.gesepoc.com<br>www.hipertensionpulmonar.es<br>www.sefq.es www.ecfs.eu<br>www.taitest.com	GesEPOC®<br>Inhaladores®<br>Inhalapp®<br>AsmaControl®<br>ForoAsma®<br>FISSIOS®<br>ICODEX®<br>CheckEPOC®<br>iDoctus®<br>Vivir con EPOC® MyTherapy®<br>VikAsma®	@SeparRespira<br>@NeumoMadrid<br>@ArchBronconeumo<br>@SEAIC_Alergia<br>@NEUMO_SEFH

Foundations and Associations available to patients

Respiratory disease	Patients' Associations and Foundations
Asthma	FENAER<br>SEPAR
Alpha-1 antitrypsin deficiency	Alfa 1 Spain
COPD	APEPOC, APEPOC Spain
Cystic Fibrosis	FEFQ
Idiopathic pulmonary fibrosis	AFEFPI
Pulmonary hypertension	FCHP, ANHP, Hpertension pulmonar España

AFEFPI: Association of relatives and patients with idiopathic pulmonary fibrosis, familiar fibrosis and related disorders, ALFA 1 Spain: Association of Spain, for patients and families affected by Alpha-1 Antitrypsin Deficiency, ANHP: Spanish National Pulmonary Hypertension Association, APEPOC: Association of Patients with COPD, FCHP: Foundation Against Pulmonary Hypertension, FEFQ: Spanish Cystic Fibrosis Federation, FENAER: Spanish Federation of Associations of Allergic Patients and Respiratory Diseases, SEPAR: Spanish Society of Pneumology and Thoracic Surgery
