# Supplementary material for: Transforming respiratory diseases management: a CMO-based hospital pharmaceutical care model
Source: Front Pharmacol. 2024 Oct 23;15:1461473. doi: 10.3389/fphar.2024.1461473 (PMC11540901; doi:10.3389/fphar.2024.1461473)
Supplement: Supplementary file 4 [file DataSheet1.PDF]

Stratification tool and pharmaceutical care model for patients with respiratory diseases	
**Respiratory disease**	
Diagnosis I	▪ Severe asthma<br>▪ Chronic obstructive pulmonary disease COPD<br>▪ Cystic fibrosis<br>▪ Non-cystic fibrosis bronchiectasis<br>▪ Idiopathic pulmonary fibrosis<br>▪ Pulmonary hypertension<br>▪ Interstitial lung diseases other than idiopathic pulmonary fibrosis<br>▪ Other (if other, please specify which one)
Diagnosis II<br>(in case there are two primary diagnoses of respiratory diseases)	▪ Severe asthma<br>▪ Chronic obstructive pulmonary disease COPD<br>▪ Cystic fibrosis<br>▪ Non-cystic fibrosis bronchiectasis<br>▪ Idiopathic pulmonary fibrosis<br>▪ Pulmonary hypertension<br>▪ Interstitial lung diseases other than idiopathic pulmonary fibrosis<br>▪ Other(if other, please specify which one)
**Demographic variables**	
Pregnant and postpartum patients	Yes (1 p) / No (0 p)
Age<br>(Variable subject to the criteria of each autonomous community)	▪ Between 0 and 15 years old, or until the age at which the patient is referred to adult consultation (use the model of children with chronic health conditions)<br>▪ Under 18 years old attended in adult consultation (Priority 1)<br>▪ Between 18 and 65 years old (0 p)<br>▪ Over 65 years old (2 p)
Body Mass Index (BMI)	▪ BMI: <18.4 kg/m² (1p; 3 p if cystic fibrosis)<br>▪ BMI: 18.4-30 kg/m²(0 p)<br>▪ BMI: ≥30 kg/m² (3 p)
**Clinical variables**	
Respiratory comorbidity<br>Patient with more than one respiratory pathology that requires medication.	Yes (2 p) / No (0 p)
Non-respiratory comorbidity<br>Patient with the presence of at least one non-respiratory pathology requiring medication. Excludes psychiatric illnesses.	Yes (3 p) / No (0 p)
Mental or behavioral disorders<br>Patient with non-transitory mental or behavioural disorders with prescription of specific drugs.	Yes (3 p) / No (0 p)
Cognitive impairment<br>Patient with presence of mild to severe cognitive-sensory impairment.<br>(Cognitive impairment is considered if there is documentation in the medical history or suspicion by the healthcare professional).	Yes (4 p) / No (0 p)
Severity of the condition<br>The patient has severe pathological involvement or requires oxygen therapy.<br>(See Appendix 4)	Yes (2 p) / No (0 p)

**Variables related to treatment**	
Lack of adherence<br>Patient with poor adherence* to any prescribed medication.	Yes (4 p) / No (0 p)
Medications that can worsen respiratory conditions<br>Patient with prescribed medications that can worsen the respiratory condition.<br>(See Appendix 5)	Yes (4 p) / No (0 p)
High alert medication<br>Patient with prescribed medications listed in the ISMP.**	Yes (4 p) / No (0 p)
Pharmacotherapeutic goals<br>Patient with unachieved pharmacotherapy goals***	Yes (2 p) / No (0 p)
Polypharmacy<br>Use of 6 or more active ingredients	Yes (3 p) / No (0 p)
f available at the center, it is recommended to assess the complexity of the medication regimen using the Medication Regimen Complexity Index (MRCI). Medication will be considered complex according to the MRCI if the patient scores higher than 11.	
Complex drugs<br>Patient with a prescription for hospital medications that require handling prior to administration and/or a device for administration (e.g., nebulizers, pen, intravenous injection, etc.).	Yes (2 p) / No (0 p)
Patient naive<br>Hospital therapy-naive patient	Yes (1 p) / No (0 p)
Changes in medication in the last 6 months<br>Changes in the regular regimen of prescribed medication in the last 6 months<br>(The consideration includes all prescribed medication, not just those related to respiratory conditions).	Yes (1 p) / No (0 p)
**Socio-Healthcare variables**	
Tobacco<br>Smoking patient	Yes (4 p) / No (0 p)
Alcohol/drugs<br>Patient with alcoholism and/or drug addiction	Yes (3 p) / No (0 p)
Occupational exposure to particulate matter<br>Patient with occupational exposure to particulate matter	Yes (3 p) / No (0 p)
Socio-economic status<br>Patient with unfavourable socio-economic conditions	Yes (3 p) / No (0 p)
A patient will be considered to have unfavorable socioeconomic conditions if their circumstances may prevent them from maintaining their medication regimen or administering treatment under appropriate sanitary and preservation conditions or maintaining healthy living conditions. This includes situations such as being homeless, socially isolated, without family support (with or without functional dependency), experiencing financial instability, and similar conditions.	
Quality of life<br>Patient with severe impairment in any of the dimensions of quality of life of the EQ-5D-5L Questionnaire.	Yes (3 p) / No (0 p)
The questionnaire should be completed when there is suspicion of severe impairment or diminished quality of life.<br>*Poor quality of life according to the EQ-5D-5L is considered to be poor if the patient has moderate or higher severity problems in any of the dimensions and/or a score of less than 50 on the numbered scale from 0 to 100.*	
**Variables related to healthcare resources**	
Hospitalizations and emergency room visits<br>The patient has had at least two admissions/visits to the emergency department, both primary care and hospital, or one admission to the ICU, due to decompensation of the respiratory pathology in the last year.	Yes (3 p) / No (0 p)

*Lack of adherence is considered if the patient is non-adherent to any prescribed medication, regardless of whether it is for the respiratory condition.
The lack of adherence should be validated using one of the following methods:

- Morisky-Green-Levine Questionnaire: Good adherence is considered if the responses are as follows: 1. No; 2. Yes; 3. No; 4. No. Any other combination is considered poor adherence. According to the Morisky-Green-Levine Questionnaire, adherence is considered low if the patient scores 6 or lower.
- Medication Possession Ratio: Definedas the sum of days supplied dispensed in a time period divided by the days in that time period. Poor adherence is considered if the Medication Possession Ratio is less than 0.8.

For patients with severe asthma and COPD, adherence will be evaluated using the Test of Adherence to Inhalers (TAI) instead of the Morisky-Green-Levine Questionnaire. Poor adherence according to the TAI is less than 45.

**ISMP (Institute for the Safe Use of Medicines) List of high-alert medications.

- Therapeutic groups

  - IV contrast agents
  - IV inotropic agents (e.g. digoxin, milrinone)
  - IV adrenergic agonists (e.g. adrenaline, dopamine, noradrenaline)
  - IV and inhaled general anaesthetics (e.g. ketamine, propofol)
  - IV adrenergic antagonists (e.g. esmolol, labetalol)
  - IV platelet antiaggregants (e.g. abciximab, eptifibatide, tirofiban)
  - IV anti-arrhythmics (e.g. amiodarone, lidocaine)
  - Oral anticoagulants (e.g. acenocoumarol, dabigatran)
  - Oral anti-diabetics (e.g. glibenclamide)
  - Neuromuscular blockers (e.g. suxamethonium, rocuronium, vecuronium)
  - Cytostatics, parenteral and oral
  - Heparin and other parenteral anticoagulants (e.g., antithrombin III, heparin sodium, enoxaparin, fondaparinux, lepirudin)
  - IV and subcutaneous insulins
  - Drugs for moderate IV sedation (e.g. midazolam, dexmedetomidine)
  - Oral drugs for moderate sedation in children (e.g. chloral hydrate, midazolam)
  - Medicines in conventional and liposomal forms (e.g. amphotericin B).
  - Medicines for epidural or intrathecal administration
  - Parenteral nutrition
  - IV, transdermal and oral opioids (all presentations)
  - Cardioplegic solutions
  - Hypertonic glucose solutions (≥20%)
  - Dialysis solutions (peritoneal and haemodialysis)
  - Thrombolytics (e.g. alteplase, tenecteplase)

- Specific medicinal products

  - Sterile water for injection, inhalation and irrigation in containers of volume ≥ 100 mL (excluding bottles)
  - Potassium chloride, IV (concentrated solution)
  - Hypertonic sodium chloride (>0.9%)
  - Epoprostenol IV
  - Potassium phosphate IV
  - Oral methotrexate (non-oncological use)
  - Sodium nitroprusside IV
  - Oxytocin IV
  - Promethazine IV

- Magnesium sulphate IV
- Tincture of opium
- Vasopressin

ISMP list of high-alert medicinal products for chronic patients

- Therapeutic groups
- Antiplatelet agents (including aspirin)
- Oral anticoagulants
- Narrow-range anti-epileptics (carbamazepine, phenytoin and valproic)
- Non-steroidal anti-inflammatory drugs
- Antipsychotics
- Benzodiazepines and analogues
- ß-adrenergic blockers
- Oral cytostatics
- Long-term corticosteroids (≥ 3 months)
- Loop diuretics
- Oral hypoglycaemic agents
- Immunosuppressants
- Insulins
- Opioids
- Specific medicines
- Amiodarone /dronedarone
- Oral digoxin
- Spironolactone /eplerenone
- Oral methotrexate (non-oncological use)

*** Include objectives related to comorbidities.

The answer will be "Yes" if the patient has not achieved the established pharmacotherapeutic goals.
The answer will be "No" if the patient has achieved the established pharmacotherapeutic goals or if such goals have not been established for them.
