# Supplementary material for: Transforming respiratory diseases management: a CMO-based hospital pharmaceutical care model
Source: Front Pharmacol. 2024 Oct 23;15:1461473. doi: 10.3389/fphar.2024.1461473 (PMC11540901; doi:10.3389/fphar.2024.1461473)
Supplement: Supplementary file 5 [file DataSheet5.PDF]

Drug that can worsen respiratory conditions

Diseases	Drug that can worsen respiratory condition
Severe asthma	• Nonselective beta blocker<br>• ACE<br>• NSAIDs
COPD	• Nonselective beta blocker<br>• ACE
CRSwNP	• Nasal decongestant > 3-5 days<br>• Beta-blockers<br>• Aspirin<br>• Chlorpromazine<br>• NSAIDs<br>• Gabapentin<br>• Alpha<br>• adrenergic receptor antagonists<br>• PDE5 inhibitors<br>• ACE<br>• Alcohol and Cocaine
Idiopathic pulmonary fibrosis and other ILDs	• Adalimumab<br>• Etanercept<br>• Mitomycin<br>• Amiodarone<br>• Infliximab<br>• Paclitaxel<br>• Bleomycin<br>• ICIs<br>• Pemetrexed<br>• Certolizumab<br>• Interferon-gamma<br>• Penicillamine<br>• Erlotinib<br>• Methotrexate

ACE: Angiotensin-converting enzyme inhibitors; COPD: Chronic obstructive pulmonary disease; ICIS: Immune checkpoint inhibitors; ILDS: interstitial lung diseases; NSAIDs: Nonsteroidal antiinflammatory drugs; PDE5: phosphodiesterase-5;
